# Novel strategies to target the ubiquitin proteasome system in multiple myeloma

**DOI:** 10.18632/oncotarget.6658

**Published:** 2015-12-18

**Authors:** Susanne Lub, Ken Maes, Eline Menu, Elke De Bruyne, Karin Vanderkerken, Els Van Valckenborgh

**Affiliations:** ^1^ Laboratory of Hematology and Immunology, Myeloma Center Brussels, Vrije Universiteit Brussel, Brussels, Belgium

**Keywords:** ubiquitin proteasome system, multiple myeloma

## Abstract

Multiple myeloma (MM) is a hematological malignancy characterized by the accumulation of plasma cells in the bone marrow (BM). The success of the proteasome inhibitor bortezomib in the treatment of MM highlights the importance of the ubiquitin proteasome system (UPS) in this particular cancer. Despite the prolonged survival of MM patients, a significant amount of patients relapse or become resistant to therapy. This underlines the importance of the development and investigation of novel targets to improve MM therapy. The UPS plays an important role in different cellular processes by targeted destruction of proteins. The ubiquitination process consists of enzymes that transfer ubiquitin to proteins targeting them for proteasomal degradation. An emerging and promising approach is to target more disease specific components of the UPS to reduce side effects and overcome resistance. In this review, we will focus on different components of the UPS such as the ubiquitin activating enzyme E1, the ubiquitin conjugating enzyme E2, the E3 ubiquitin ligases, the deubiquitinating enzymes (DUBs) and the proteasome. We will discuss their role in MM and the implications in drug discovery for the treatment of MM.

## THE UBIQUITIN PROTEASOME SYSTEM (UPS)

The UPS is responsible for regulated proteolysis and therefore plays a key role in different biological processes such as cell cycle progression, receptor down-regulation, gene transcription and apoptosis by selectively targeting cellular proteins for degradation [1]. This process includes two specific and sequential steps: ubiquitination and proteasomal degradation.

### Ubiquitination

**Figure 1:**
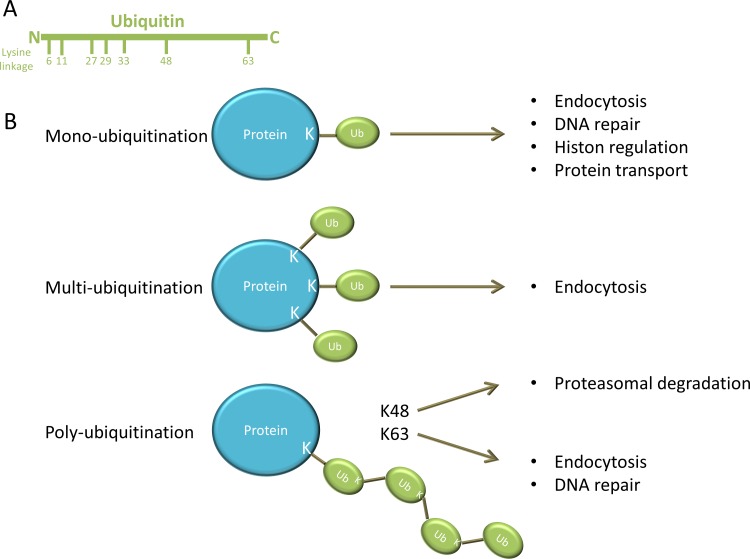
different forms of ubiquitin modification **A.** Ubiquitin is composed of 76 amino acids and contains seven lysine residues on which other ubiquitin molecules can be conjugated. **B.** A single ubiquitin is linked to a lysine (K) residue on the protein during mono-ubiquitination. Mono-ubiquitination is involved in endocytosis, DNA repair, histon regulation and protein transport. Multi-ubiquitination is obtained when multiple single ubiquitins are linked to different lysine residues on the protein and is implicated in endocytosis. However poly-ubiquitination is obtained when a poly-ubiquitin chain is linked to the protein. Poly-ubiquitin chains formed on lysine 48 (K48) of ubiquitin have a well-known role in targeted protein degradation by the 26S proteasome, whereas poly-ubiquitin chains formed through lysine 63 (K63) are involved in DNA repair and endocytosis.

**Figure 2:**
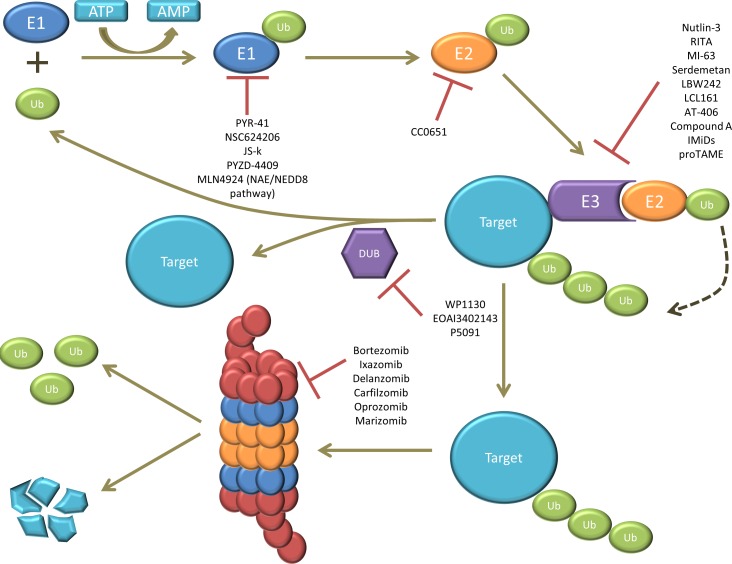
illustration of the ubiquitin proteasome system (UPS) The ubiquitin (Ub) is first activated by E1 in an ATP-dependent manner. The activated ubiquitin is then transferred to E2. E3 forms a complex with the target protein (or substrate) and recruits the E2-ubiquitin complex. The activated ubiquitin is then transferred from E2 to the target protein. This ubiquitination process can be reversed by deubiquitinating enzymes (DUBs). Finally, the ubiquitinated target gets recognized and degraded by the 26S proteasome. Several compounds targeting E1, E2, E3, DUBs and the proteasome have been studied, from which some of them are currently approved for the treatment of MM, while others are still under (pre)-clinical investigation.

Ubiquitination is a stepwise cascade of enzymatic reactions and requires the ubiquitin activating enzyme (E1), ubiquitin conjugating enzyme (E2) and the ubiquitin ligase (E3). First E1 activates ubiquitin in an ATP-dependent manner and connects it to E2, which subsequently forms a complex with E3 and the target protein (or substrate) [2, 3]. The activated ubiquitin is then transferred to a lysine (K) residue on the target protein. Ubiquitin is a small protein composed of 76 amino acids and contains seven lysine residues on which other ubiquitin molecules can be conjugated (Figure 1A). As illustrated in Figure 1B: the substrate protein can be modified by: i) mono-ubiquitination: a single ubiquitin is linked to the protein; ii) multi-ubiquitination: multiple single ubiquitins are linked to the protein or iii) poly-ubiquitination: a poly-ubiquitin chain is linked to the protein [4]. The type of ubiquitination determines the fate and functional outcome of the protein. Mono-ubiquitination is involved in different cellular processes such as endocytosis, DNA repair, histon regulation and protein transport. Multi-ubiquitination is also implicated in endocytosis. By contrast, poly-ubiquitin chains formed on lysine 48 (K48) of ubiquitin have a well-known role in targeted protein degradation by the 26S proteasome, whereas poly-ubiquitin chains formed through lysine 63 (K63) are involved in DNA repair and endocytosis [1, 2, 4, 5]. This ubiquitination process can be reversed by deubiquitinating enzymes (DUBs). The balance between ubiquitination and deubiquitination activities regulates the level and activity of the protein substrates and thus cell homeostasis [2]. A schematic overview of the ubiquitination process is presented in Figure 2.

### Proteasomal degradation

**Figure 3:**
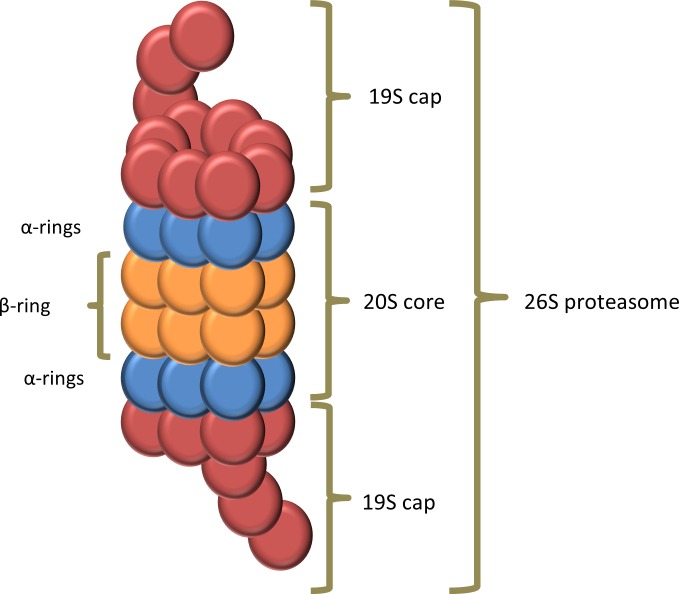
composition of the proteasome The proteasome consists of a 20S core particle and two 19S regulatory particles. The 20S core particle is composed of four rings creating a cylinder where the proteolysis occurs. The two α-rings are each formed by seven distinct α-subunits and serve as a docking domain for the 19S regulatory particle. The catalytic chamber is formed by the two inner β-rings, which are each formed by seven distinct β-subunits. The 19S regulatory particle binds a polyubiquitin chain and cleaves and recycles the ubiquitin. The substrate is then denaturated or unfolded and fed into the catalytic chamber where it is degraded into small peptides.

Proteins with a poly-ubiquitination at K48 of the ubiquitin will be degraded by the 26S proteasome [4]. The 26S proteasome also known as the “proteasome” is a large (more than 2000 kDA) multi-protein complex present in the nucleus and cytoplasm of all eukaryotic cells [4, 6]. It is composed of one 20S core particle and two 19S regulatory particles. The 20S core particle is composed of four rings creating a proteolytic cylinder. The two outer rings, also called α-rings, are each formed by seven distinct α-subunits and serve as a docking domain for the 19S regulatory particle. The two inner β-rings are each formed by seven distinct β-subunits and are responsible for proteolysis. Three of these β-subunits (β1, β2 and β5) contain a catalytic activity with specific substrate specificity: a chymotrypsin-like, trypsin-like and peptidylglutamyl-peptide hydrolyzing activity, respectively [7]. In Figure 3 the composition of the proteasome is illustrated. The 19S regulatory particle binds a polyubiquitin chain and cleaves it from the substrate and recycles the ubiquitin. The substrate is then denaturated/unfolded and subsequently degraded into small peptides [6]. Interestingly, Avram Hershko, Aaron Ciechanover and Irwin Rose received a Nobel prize in chemistry for the discovery of ubiquitin-mediated protein degradation, emphasizing the importance of this pathway in cell physiology [8].

### Ubiquitin-like proteins (UBLs)

In the early eighties the ubiquitin-like proteins (UBLs) were discovered [9]. UBLs are molecules that modify molecular targets in a similar way as ubiquitin. All the UBLs have basically the same three-dimensional structure and are attached to substrates via related enzymatic pathways as ubiquitin. The UBLs conjugation pathways have their own specific E1, E2, E3 and deconjugating enzymes. UBLSs such as NEDD8, SUMO, ISG15 and Atg8 regulate many cellular processes including transcription, DNA repair, signal transduction, autophagy, and cell-cycle control [9, 10]. Therefore it is not surprising that the (mal)function of UBLs are associated with various human diseases. There is a functional crosstalk between the UBL and ubiquitin conjugating pathway. For instance, the activation of the cullin-RING ligases (CRLs), an E3 ubiquitin ligase, requires the modification of cullin with NEDD8 [11, 12].

## TARGETING THE UBIQUITIN PROTEASOME SYSTEM IN MM

### Multiple myeloma

Multiple myeloma is a malignant disease of neoplastic plasma cells with a strong tropism for the bone marrow (BM) and is characterized by the presence of monoclonal immunoglobulins in the blood and/or urine [13]. The disease typically involves end-organ damage also referred to as the CRAB criteria: hyperCalcemia, Renal failure, Anemia and Bone lesions. MM is characterized by the presence of M-protein in serum or urine, more than 10% monoclonal plasma cells in the BM and the presence of end-organ damage [14]. MM is the second most common hematological malignancy and accounts for 1% of all cancers and 13% of all hematological cancers [15]. In 2012, the incidence rate per 100.000 citizens was 5.3 and 3.8 in respectively Belgium and Europe [16]. It primary affects elderly people with a median age of 65 years at diagnosis and is slightly more common in men than in women [17]. There are racial differences observed in the incidence of MM. It is twice as common in African Americans compared to Caucasians which makes it the most frequent hematological malignancy in this racial group [13, 18]. MM usually evolves from an asymptomatic plasma cell dyscrasia called monoclonal gammopathy of undetermined significance (MGUS) [19]. MGUS is defined by the presence of serum M-protein (< 3g/dL), less than 10% clonal plasma cell in the BM and importantly the absence of the typical MM-related end-organ damage [20]. The risk of MGUS patients to progress to MM (or other related diseases) is approximately 1% per year [19, 21]. In some patients MM is preceded by an intermediate, asymptomatic but more advanced stage known as smoldering MM (SMM). SMM is characterized by a high serum M-protein (≥ 3g/dL), ≥ 10% monoclonal plasma cells in the BM and importantly the absence of the typical MM-related end-organ damage [22]. Up till now SMM patients were closely monitored until the disease progresses to symptomatic MM, after which treatment was initiated [15, 22]. However, recently the International Myeloma Working Group (IMWG) added additional features as criteria of clinical diagnosis of treatment requiring MM. This other myeloma defining events were associated with an 80% risk of disease progression within 2 years and include: clonal BM plasma cell percentage ≥ 60; an involved/uninvolved free light chain (FLC) ratio of ≥100; more than one focal BM lesion detected by MRI. This novel myeloma defining events identify a subset of patients previously viewed as SMM that are now considered as MM [23]. This was based on a recent study that demonstrated that treatment of high-risk SMM with lenalidomide/dexamethasone delays progression to active disease and increases overall survival [24].

In some advanced cases, MM cells become independent of the BM stroma cells which results in extramedullary disease and secondary plasma cell leukemia [25]. The BM microenvironment plays an important role in the survival and progression of MM cells. The BM niche consists of cell-cell and cell-matrix interactions, growth factors and cytokines. Osteoclasts, osteoblasts, endothelial cells, fibroblast, immune cells and adipocytes all constitute to the cellular compartment of the BM. A complex network of fibronectin, laminin and collagen form the extracellular compartment. There is a bidirectional signaling loop between MM and the BM microenvironmental cells, thereby promoting tumor proliferation and growth, drug resistance, homing of the MM cells, angiogenesis and bone lesions [13, 26]. MM is a very heterogeneous disease with variable clinical and biological characteristics, response to treatment and survival outcomes among patients. This has mainly been attributed to alterations in molecular characteristics of the plasma cells. Some recurrent genetic abnormalities can be found in MM such as hyperdiploidy (associated with multiple trisomies), loss of chromosome 13, translocations affecting the Ig heavy chain (IgH) locus (14q32) [27, 28].

An essential part of the management of MM disease is high-dose therapy (melphalan) followed by autologous stem cell transplantation. MM patients are considered eligible for transplantation when they are younger than 65-70 years, their organs are functioning adequate and they have no comorbidities. A major advance in the treatment of MM this past two decades is the use of novel drugs like the immunomodulatory drugs (thalidomide and lenalidomide) and the proteasome inhibitors (bortezomib and carfilzomib) as frontline therapy in transplant as well as in non-transplant candidates. The introduction of these agents have considerably improved complete response rates, time to progression, progression-free survival and overall survival [13, 15].

### Targeting proteasomal degradation in MM: proteasome inhibitors

The final step in the UPS pathway is the protein degradation by the proteasome. The proteasome was the first element of the UPS demonstrated to be a therapeutic target. Proteasome inhibitors were first created to investigate the function of the proteasome catalytic activity but were soon to be found interesting agents for cancer therapy [2]. MM cells were particularly found to be susceptible to proteasome inhibition because they are proliferative, they over-produce defective proteins which need to be degraded by the proteasome and they up-regulate signaling pathways dependent on the 26S proteasome [29]. Different proteasome inhibitors are currently used for the treatment of MM and several new generation proteasome inhibitors are under clinical investigation. Here below we will discuss the different classes of proteasome inhibitors.

#### Boronates

Bortezomib is a dipeptide boronic acid proteasome inhibitor. It reversibly inhibits the chymotrypsin-like activity of the 20S proteasome. The first *in vitro* study with bortezomib in MM demonstrated that numerous cellular processes were affected by the accumulation of intracellular proteins. Moreover bortezomib treatment of MM inhibited growth, induced apoptosis and overcame drug resistance [30]. The anti-tumor effect of bortezomib has been attributed to alterations of the NF-κB activity [31], accumulation of cell cycle proteins [30], a disturbed balance between pro- and anti-apoptotic proteins [32, 33], stimulation of endoplasmic reticulum stress [34], and impairment of the DNA repair pathway in the MM cells [35]. Bortezomib also inhibited the paracrine growth of MM cells by decreasing the adherence of MM cells to BMSCs and inhibiting NF-κB dependent IL-6 secretion by the stromal cells [30]. Moreover bortezomib also induced apoptosis in endothelial cells and decreases VEGF secretion, resulting in reduced angiogenesis [36]. Osteoblast differentiation and activity increased upon bortezomib treatment leading to increased bone formation [37]. These preclinical studies demonstrated that bortezomib could be promising in especially MM and therefore phase 1-3 clinical trials were quickly initiated [38-41]. In 2003, bortezomib was FDA approved for the treatment of relapsed/refractory MM [42]. Later on, it was approved for relapsed and newly diagnosed MM patients in respectively 2005 and 2008 [41, 43]. Although bortezomib significantly improved the survival of MM patients, there are still some challenges to overcome. First of all, bortezomib is associated with peripheral neuropathy in 37-44% of the MM patients. Bortezomib-induced peripheral neuropathy (BIPN) can really affect the quality of life of the patient due to the severe pain. There have been many attempts to manage the BIPN, such as co-treatment with the heat shock protein inhibitor tanespimycin which appears to reduce the incidence of BIPN [44]. Moreover subcutaneous instead of intravenous administration of bortezomib has shown to reduce the incidence of BIPN [45]. Also the second generation proteasome inhibitors carfilzomib and NPI-0052 showed reduced incidence of peripheral neuropathy [44]. A second challenge is the fact that bortezomib is not universally effective. Not all patients are responsive and the responders eventually relapse [46]. This has led to many clinical trials in MM combining bortezomib with other agents to enhance efficacy [47].

Ixazomib citrate (MLN9708) is the first oral proteasome inhibitor under clinical investigation in MM. MLN9708 is also a boronate proteasome inhibitor but with a different physicochemical profile. MLN9708 (ixazomib citrate) is directly hydrolyzed in plasma to the biologically active form MLN2238 (ixazomib). MLN2238 preferentially and reversibly inhibits the β5 chymotryptic-like subunit of the proteasome with similar potency and selectivity as bortezomib; but it has a significantly shorter dissociation half-life. This shorter half-life is thought to improve tissue distribution [48]. Ixazomib has potent *in vivo* and *in vitro* anti-MM effects and has evidenced clinical anti-MM activity in patients [49-52]. In Phase 1/2 clinical studies ixazomib had a good safety profile with limited peripheral neuropathy. These trials showed that ixazomib exerted anti-MM activity as a single agent in relapsed/refractory MM and in combination with lenalidomide and dexamethasone in newly diagnosed patients [50, 52]. Ixazomib is currently entering phase 3 clinical trial for the treatment of MM (https://clinicaltrials.gov).

Delanzomib (CEP-18770) is an orally bioavailable boronic-acid containing proteasome inhibitor that similar to bortezomib reversibly inhibits the chymotrypsin activity of the proteasome. Delanzomib has potent *in vitro* anti-MM effects as a single agent and in combination with bortezomib or melphalan [53, 54]. Importantly delanzomib showed favorable cytotoxicity against other cell types from the BM, inhibited angiogenesis and repressed RANKL-induced osteoclastogenesis [54]. In different *in vivo* studies delanzomib reduced tumor growth as a single agent or in combination with bortezomib, melphalan, lenalidomide and dexamethasone [53-55]. Delanzomib showed a favorable safety profile with lack of neurotoxicity in relapsed/refractory MM patients during a phase 1 trial. However a dose-limiting skin rash was observed in approximately half of the patients [56]. A second phase 1/2 trial has been started but terminated due to unmanageable toxicity [57].

#### Epoxyketones

Carfilzomib is a tetrapeptide epoxyketone that unlike bortezomib irreversibly binds and selectively inhibits the chymotrypsin-like activity of the 20S proteasome leading to a more sustained proteasome inhibition [58]. Carfilzomib has shown to induce apoptosis and growth arrest in human MM cells [58, 59]. Moreover it was effective in bortezomib resistant MM cells and acted synergistically with dexamethasone to induce cell death [59]. Carfilzomib has been FDA approved under accelerated procedure in 2012 for the treatment of MM patients who received at least two prior therapies, including bortezomib and an immunomodulatory drug, and who have disease progression on or within 60 days after the completion of the last therapy [60]. Currently carfilzomib is under extensive investigation in different clinical trials in relapsed/refractory and previously untreated MM and many other cancer types (https://clinicaltrials.gov).

Because of the poor oral bioavailability of carfilzomib, it is administrated intravenously. To increase the flexibility of dosing and the convenience for patients an orally bioavailable derivate of carfilzomib, oprozomib (ONX0912) was developed [61]. Oprozomib is an epoxyketone-based proteasome inhibitor and was as potent as carfilzomib to kill MM cells. Moreover oprozomib increased the anti-MM activity of bortezomib, lenalidomide, dexamethasone, and a pan-histone deacetylase inhibitor. Animal studies showed that oprozomib was able to reduce tumor progression and increase survival [62]. In addition to the anti-MM effect, oprozomib decreased the myeloma-associated bone disease [63]. Currently oprozomib is in phase 1/2 trial for relapsed/refractory, relapsed and newly diagnosed MM patients as single agent or in combination with other anti-MM drugs (https://clinicaltrials.gov).

#### Salinosporamides

The proteasome inhibitors carfilzomib and oprozomib are peptides and therefore susceptible for degradation by endogenous proteases and peptidases in the plasma, reducing their bioavailability. The bioavailability could be increased by non-peptidic proteasome inhibitors such as marizomib (NPI-0052) [61]. Marizomib is an oral β-lactone proteasome inhibitor derived from the marine bacterium Salinospora tropica and irreversibly binds to the three catalytic activities of the proteasome [64, 65]. Marizomib is effective in bortezomib sensitive and resistant MM cell lines and in an *in vivo* mouse model [65]. Moreover it acts synergistically with bortezomib and lenalidomide [66, 67]. Marizomib is currently in phase 1/2 clinical trial for the treatment of relapsed or relapsed/refractory MM (https://clinicaltrials.gov).

Despite the promising clinical advances, most of the MM patients relapse and die of the disease. This highlights the importance of the development and investigation of novel targets and treatment options for MM patients. The increased survival of MM patients by the addition of the proteasome inhibitor bortezomib to the treatment, underlines the importance of the UPS in MM cells. An emerging and promising approach is to target more disease specific components of the UPS to reduce side effects and overcome resistance. The following section will link UPS components with MM and emphasize their therapeutic potential.

## UBIQUITIN AND DEUBIQUITINATING ENZYMES IN MM: NOVEL THERAPEUTIC OPPORTUNITIES

### E1 ubiquitin activating enzymes

The first step in the ubiquitin conjugation pathway is the activation of ubiquitin in an ATP-dependent manner. Initially E1 ubiquitin activating enzyme binds ATP and adenylates the C-terminal glycine of ubiquitin, which then forms a covalent enzyme-ubiquitin thioester bond between the catalytic cysteine of E1 and ubiquitin [68]. Currently there are two human ubiquitin E1 enzymes identified, UBA1 and UBA6, of which UBA1 is the principle isoform for protein degradation [69, 70]. Knockdown of E1 resulted in a decreased viability of MM and leukemia cells suggesting that it could be an interesting target [70]. There are three possible approaches to inhibit ubiquitin activation. First the binding of ATP to the E1 enzyme may be blocked by an ATP-competitive small molecule inhibitor. Second the binding of ubiquitin to E1 could be blocked by targeting the active thiol site. Finally the E1-E2 interaction can be blocked [68, 71]. Up till now four inhibitors of E1 ubiquitin activating enzyme have been identified, Pyr-41, NSC624206, JS-K and PPZD-4409 [70, 72-74]. Pyr-41 and NSC624206 were initially discovered as inhibitors of respectively p53 and p27 ubiquitination and specifically block ubiquitin-thioester formation [73, 74]. JS-K is a nitric oxide (NO) prodrug that releases NO when metabolized by gluthatione S-transferase. It inhibits the ubiquitin-E1 thioester formation by binding of NO to the active cysteine residue on E1. In addition, it also seems to affect other signaling pathways like MAPK, Wnt and β-catenin/TCF signaling pathway [72, 75-77]. PPDZ-4409 is structurally related to Pyr-41 and showed similar E1 inhibitory effects [70]. To our knowledge Pyr-41 and NSC624206 have so far not been tested in MM. JS-K has been described to induce DNA double-strand breaks, activate the DNA damage response pathway and induce apoptosis in human MM cells *in vitro* and *in vivo* [78]. Moreover, in another study, JS-K demonstrated anti-angiogenic activity *in vitro* and *in vivo* [79]. Whether the effect of JS-K on MM cells is due to E1 inhibition has not been studied yet. PYZD-4409 has been shown to induce cell death in MM cell lines and preferentially inhibited the growth of leukemia cells over normal hematopoietic cells. The underlying mechanism of the observed cell death has been attributed to ER stress, but this was only studied in leukemia cells [70]. These studies highlight that the E1 enzyme could be a novel therapeutic target in the treatment of MM, however, the underlying mechanisms should be confirmed.

### NEDD8 activating enzyme (NAE)

The activity of the cullin-RING ligases (CRLs), a family of E3 ubiquitin ligases, is regulated by the covalent attachment of NEDD8. The neddylation of proteins is mediated by an enzymatic process similar to the ubiquitin conjugating pathway. The first step is the activation of NEDD8 by NEDD8 activating enzyme (NAE). Consequently inhibiting the NAE indirectly inhibits the activation of the CRL E3s [12, 80, 81]. CRL E3s play an important role in many biological processes such as cell cycle progression, apoptosis, signaling transduction and DNA replication. Known substrates of the CRL E3s are oncoproteins (Myc), tumor suppressors such as p21, p27, cell cycle promoters like cyclin D/E, regulators of apoptosis including Mcl-1 and signaling molecules such as the NF-κB inhibitor IκB [80]. Recently a novel small molecule inhibitor of the NAE, MLN4924, has been discovered. The NAE enzymatic activity is blocked by the binding of MLN4924 to NAE that creates a covalent NEDD8-MLN4924 adduct, preventing subsequent intraenzyme reactions [82]. The observation that MM patients with high NEDD8 transcript levels had a shorter progression free survival upon bortezomib treatment emphasizes the importance of therapeutically targeting the NEDD8 pathway. Treatment of MM cell lines and primary cells with MLN4924 resulted in an upregulation of known targets of the NEDD8 pathway and a decreased viability of MM cells. MLN4924 was as potent in a bortezomib-resistant cell line as in the parental cell line [83]. Its cytotoxic effect was assigned due to increased REDD1 expression leading to the suppression of AKT and mTOR signaling pathways [81]. Two studies demonstrated that combining MLN4924 with bortezomib enhanced the cytotoxic effect of MLN4924 [81, 83]. Interestingly, cells resistant to bortezomib due to elevated CKS1B expression were sensitive to MLN4924 through the stabilization of p21 [84]. MLN4924 has shown its potency in different MM *in vivo* models and is currently in phase 1 trial for MM patients and other malignancies (https://clinicaltrials.gov).

### E2 conjugating enzymes

To date, 35 active E2 ubiquitin-conjugating enzymes (UBC) have been described in humans. E2s are characterized by the presence of an ubiquitin-conjugating domain (UCD). E1 transfers the activated ubiquitin E2 through the thioester bond on the catalytic cysteine on E2. Next the ubiquitin is transferred to the substrate with help of the E3 ubiquitin ligase. E2s determine the fate of the proteins by regulating whether the protein is multi-, mono- or polyubiquitinated and on which site ubiquitin is conjugated. There are several studies suggesting that E2s are involved in cancer and other diseases [85]. The UBC CDC34 (also known as UBC3) is highly expressed in primary MM cells and MM cell lines compared to normal cells. CDC34 regulates the ubiquitination of proteins involved in cell cycle regulation, such as p27, a cell cycle inhibitor. Blocking CDC34 by the use of a dominant-negative strategy enhanced the cytotoxic effect of bortezomib, dexamethasone and 2-methoxyestradiol in MM cells. Moreover, IL-6 mediated protection against dexamethasone-induced cell death was abrogated by blocking CDC34 [86]. CC0651, a small molecule inhibiting CDC34, inhibited proliferation of human cancer cell lines [87]. Since the study of Chauhan et al. [86] demonstrated enhanced cytotoxicity of anti-MM agents when blocking CDC34, combining CC0651 with commonly used anti-MM agents could be a promising new treatment approach.

### E3 ubiquitin ligases

The E3 ubiquitin ligases mediate the last step of the ubiquitination pathway. Because E3 interacts both with E2-Ub and the substrate to be ubiquitinated, they determine the selectively of the ubiquitination process [68]. In humans there are about 1000 E3s that can be divided into three types characterized by their conserved structural domain and substrate recognition [88]. The first and largest type of E3s are the really interesting new gene (RING) finger family. The RING finger E3 functions as an adapter of the E2-Ub thioester and the substrate and catalyzes the transfer of ubiquitin from the E2 enzyme to the substrate [71, 89]. RING finger E3s can exist and act as a single protein such as human double minute 2 (HDM2) or be part of a multisubunit compex such as the anaphase promoting complex/cyclosome (APC/C) [88, 90]. The second type of E3, the homology to E6AP C terminus (HECT) family E3s, ubiquitinate substrates in two steps: first ubiquitin is transferred from E2 to E3 and then from E3 to the substrate [71, 89]. The U-box E3s, a third type, act as adopter proteins that recruit the E2 enzyme and the substrate for ubiquitin transfer just as RING finger E3s [90]. Some E3s have been linked to the pathogenesis of MM. In the next section we will discuss these E3s and their small molecule inhibitors in MM.

#### Human double minute 2 (HDM2)

HDM2 (also known as mouse double minute 2, MDM2), is an E3 ubiquitin ligase responsible for the proteasomal degradation and inhibition of the transcriptional activation of wild-type p53 (wt-p53), a tumor suppressor protein [91]. HDM2 is highly and constitutively expressed in MM cell lines, in cells of patients with plasma cell leukemia but not in mononuclear cells from normal BM. This overexpression has been shown to contribute to growth and survival of MM cells [92]. Several HDM2 inhibitors are identified the last years.

#### Nutlin-3

The first reported HDM2 inhibitor, nutlin-3 is a cis-imidazole analog with a strong affinity for the p53-binding pocket of HDM2. So, nutlin-3 binds to HDM2 thereby inhibiting the interaction between HDM2 and p53 and resulting in an accumulation of p53 and activation of the p53 signaling pathway [91, 93]. Therapeutic activation of p53 requires wt-p53. Since mutations or deletions of p53 are rarely detected at diagnosis of MM, HDM2 inhibition could be particularly interesting for MM patients [94-97]. Indeed, it has been demonstrated that nutlin-3 treatment of primary MM samples and cell lines with or without the presence of bone marrow stromal cells (BMSC), resulted in apoptotic cell death. Moreover the p53 pathway was reactivated in wt-p53 cells by the transcription of the downstream targets such as p21 and HDM2. In MM cell lines with mutant p53, no reactivation of the p53 pathway was observed upon nutlin-3 treatment [97, 98]. Apoptosis induced by nutlin-3 was associated with increased expression of downstream transcriptional targets of p53 such Puma, Bax and Bak. However, also p53 transcriptional independent pathways are activated [98]. Importantly, nutlin-3 was not toxic for normal BMSCs [97]. Taken together these data demonstrate that the nongenotoxic activation of the p53 pathway could be a potential new treatment strategy for MM patients. This is strengthened by the fact that nutlin-3 acts synergistically with anti-MM drugs currently used in the treatment of MM such as bortezomib and melphalan [97, 99, 100]. For the potential clinical use of nutlin-3, we have to take into account that nutlin-3 treatment can cause the acquisition of somatic mutations in p53. Therefore prolonged treatment with nutlin-3 could potentially lead to resistance to the drug [101].

#### RITA

Another p53-HDM2 interaction inhibitor has been identified in 2004 by Issaeva et al. named RITA (reactivation of p53 and induction of tumor cell apoptosis). RITA prevents the p53-HDM2 interaction by binding to p53 resulting in an accumulation of p53, expression of p53 target genes and induction of apoptosis in different cancer cell lines [102]. RITA has shown potent anti-MM activity, it activates the p53 pathway and induces apoptosis in MM cell lines and primary MM samples with wt-p53. The activation of the p53 pathway was accompanied with upregulation of the proapoptotic protein Noxa, down-regulation of the anti-apoptotic protein Mcl-1 and cleavage of caspase-3 and -8. These data were further validated in a mouse xenograft model of MM. A clear tumor regression and increase in survival was observed when these mice were treated with RITA. Moreover combination of RITA with nutlin-3 synergistically inhibited the growth of MM cells [103]. However important to mention is that the activities of RITA are not restricted to p53. Although HDM2 inhibitor resistant cells have mutations in p53, RITA was able to induce a G2/M arrest and upregulated p53 targets like HDM2, Puma, Noxa and PARP cleavage [104]. A recent study demonstrated that RITA was efficient against p53 mutated MM cells independently of the p53 pathway [105]. This is in line with the discovery of the novel role of RITA as activator of the JNK pathway. Combining RITA with the JNK activator dexamethasone resulted in a synergistic anti-MM effect [106]. This compound could function as a multi-target molecule and provides the rationale for further clinical investigation of RITA as a single agent or in combination with dexamethasone for the treatment of MM patients [106].

#### MI-63

Similar to nutlin-3, MI-63 binds to the p53 binding site of HDM2 in cells with wt-p53 [107]. In MM, it has been shown that MI-63 was effective in the induction of apoptosis and activation of p53-mediated cell death. Moreover MI-63 was able to overcome adhesion-mediated resistance, lenalidomide resistance and enhances the anti-MM effect of bortezomib and lenalidomide. At higher concentrations MI-63 was also able to induce apoptosis in cells with mutant p53 and this was associated with autophagy. The combination of MI-63 with the BH3-mimetic, ABT-737 (Bcl-2 and Bcl-xl inhibitor), had synergistic anti-MM effect both in MM cells with mutant or wt-p53. Since MI-63 showed potency *in vivo* and in primary plasma cells from patients with newly diagnosed and relapsed-refractory MM, further clinical investigation is desirable [108].

#### JNJ-26854165 (Serdemetan)

Serdemetan, another HDM2-p53 inhibitor, specifically binds to the ring domain of HDM2, thereby inhibiting the binding of the HDM2-p53 complex to the proteasome and preventing the degradation of p53 [109]. In MM, serdemetan was able to inhibit proliferation and induce an S-phase arrest, both in cells with wt and mutant p53. Caspase-3 activation was mainly observed in cells with wt-p53 but caspase-3 was also activated in cells with mutant p53 although at a lesser extent. It was shown that serdemetan exerted its anti-MM effect through inhibition of cholesterol transport by degradation of the ABCA1 transporter [110]. Currently serdemetan is in phase 1 clinical trial for advanced stage and/or refractory solid tumors (https://clinicaltrials.gov).

#### Inhibitor of apoptosis proteins (IAPs)

IAPs are a family of endogenous inhibitors of programmed cell death and are characterized by the presence of one to three baculovirus IAP repeat (BIR) domains [111]. Eight human homologues of IAPs have so far been identified, NAIP (Baculoviral IAP repeat-containing protein 1), XIAP (X-chromosome linked IAP), cIAP1 (cellular IAP1), cIAP2 (cellular IAP2), ILP2 (IAP like protein), BRUCE, survivin and livin [112]. Some family members (cIAP1, cIAP2, XIAP, ILP2 and livin) also contain a RING finger domain allowing them to ubiquitinate and degrade caspases and SMAC. Moreover it allows them to autoubiquitinate themselves resulting in their degradation [113]. In apoptotic cells, the pro-apoptotic protein SMAC is released in the cytosol and binds to the IAPs. This prevents the interaction with caspases and results in cell death [114-116]. Alterations of IAPs have been frequently reported in various human cancers, including hematological malignancies, leading to aberrant apoptosis-signaling pathways [117]. These alterations contribute to tumor cell survival, chemo-resistance, disease progression and poor patient prognosis [118]. IAPs are therefore considered as potential therapeutic targets and different inhibitors of IAPs have been developed in recent years. In MM, expression of cIAP1, cIAP2 and XIAP has been correlated with poor outcome and associated with drug resistance [119]. Three different SMAC mimetics have so far been preclinically tested in MM. Chauhan et al. demonstrated that the SMAC mimetic LBW242 induced apoptosis in cells resistant to conventional therapy. This LBW242-induced apoptosis is associated with cleavage of caspase-3, -8, -9 and PARP. Combining LBW242 with TRAIL, bortezomib and melphalan resulted in an additive/synergistic anti-MM effect. Moreover LBW242 was cytotoxic against primary MM cells from patients but not against normal lymphocytes and BMSCs. Importantly, LBW242 inhibited tumor growth and prolonged survival in a human xenograft mouse model [120]. Another SMAC mimetic, LCL161 was only cytotoxic in a subset of MM cell lines. It was shown that the resistance to LCL161 was due to the lack of cIAP2 down-regulation upon LCL161 treatment. Moreover the Jak2/Stat3 pathway was upregulated in MM cells resistant to LCL161. The combination of LCL161 and a Jak2 specific inhibitor resulted in a synergitic anti-MM effect in cell lines and in patient cells. Additionally, LCL161 sensitized cells to death inducing ligands such as TRAIL and FasL [121]. We studied the role of cIAP2 in TRAF3 deleted/mutated MM cell lines and found that overexpression of cIAP2 was associated with resistance to proteasome inhibitors [122]. In addition the SMAC mimetic AT-406 sensitized MM cells to the proteasome inhibitors bortezomib and carfilzomib. Taken together these data provide evidence for further clinical evaluation of SMAC mimetics, alone and in combination, especially in MM patients with TRAF3 deletion/mutation [122]. Currently LCL161 is in phase 2 clinical trial for patients with MM and breast cancer. AT-406 is in phase 1 clinical trial for patients with solid tumors, lymphoma or acute myelogenous leukemia (AML) (https://clinicaltrials.gov).

#### Skp1-Cullin-Fbox complex (SCF-complex)

The SCF-complex type E3 ubiquitin ligase is a multiprotein complex consisting of three core components (Skp1, Cullin-1, Roc1) and one variable component, the F-box protein. The F-box protein is considered as a potential target with great specificity since they function as a substrate receptor and thereby determine the substrate to be ubiquitinated. So far 69 F-box proteins have been identified [123, 124]. Skp2 is an F-box protein that regulates the ubiquitination of several targets such as, p27^Kip1^, p21^Cip1^ and p57^Kip2^. P27 is an important cell cycle regulator that induces a cell cycle arrest and inhibits the G_1_/S transition [125-127]. In MM it has been shown that patients with low p27 expression had a shorter overall survival, suggesting that targeting the SCF^Skp2^ complex to restore p27^Kip1^ levels could be a new treatment strategy in MM [128, 129]. Inhibition of the SCF^Skp2^ with Compound A (CpdA) in MM cells resulted in accumulation of the SCF^Skp2^ substrate p21 without activating the heat shock protein response. Moreover CpdA treatment resulted in a G_1_/S cell cycle arrest and SCF^Skp2^ and p27 dependent cell death. This observed cell death was caspase independent and mediated through activation of autophagy. CpdA was able to overcome drug resistance to conventional anti-MM drugs and even acted synergistically with bortezomib. Importantly, the anti-MM effect of CpdA was confirmed on primary MM cells of patients. Other cells from the BM compartment were not affected by CpdA [130]. These data indeed provide evidence for further clinical evaluation of CpdA in the treatment of MM.

Another F-box protein is Fbxo9 and has shown to be highly expressed in MM cells compared to normal plasma cell in 30% of the cases. Higher expression of Fbxo9 in MM patients correlated with a higher progression-free survival and better response to bortezomib, confirming the idea that in these cases Fbxo9 promotes survival of MM cells. Moreover knockdown of Fbxo9 resulted in apoptosis in MM cells with high Fbxo9 expression. This study underscores the potential use of Fbxo9 as a target in MM with high Fbxo9 expression and suggests that Fbxo9 could be useful in predicting clinical response to proteasome inhibition [131]. To our knowledge no Fbxo9 inhibitors have been identified.

#### Cereblon

Currently the standard treatment regimen of MM includes the immunomodulatory drugs (IMiDs) thalidomide or the more novel analogues lenalidomide or pomalidomide [13]. Thalidomide was initially used for hyperemesis gravidarum in pregnant women but was found to cause serious birth defects [132]. At that time the working mechanism was largely unknown but recent studies demonstrated that the specific target for thalidomide is the E3 ubiquitin ligase cereblon (CRBN) [133]. It was confirmed that CRBN was the principal target for the observed anti-MM effect of the IMiDs [134, 135]. Depletion of CRBN caused cell death in human MM cells, but a subset of cells survived despite stable depletion of CRBN. These cells have been found to be resistant to lenalidomide and pomalidomide but sensitive to other anti-MM agents such as bortezomib and melphalan. In MM cells resistant to lenalidomide they found an acquired deletion of CRBN. Moreover, the genetic changes upon lenalidomide treatement were drastically abolished when CRBN was depleted, suggesting that CRBN is essential for the anti-MM effect of lenalidomide. Importantly patients with lenalidomide resistance had reduced CRBN expression levels [135]. Taken together, CRBN is required for the anti-MM activity of lenalidomide and moreover could be used as a biomarker for clinical evaluation of anti-MM efficacy. Currently many combinations of immunomodulatory drugs with other agents are under clinical evaluation (https://clinicaltrials.gov).

#### Anaphase promoting complex /cyclosome (APC/C)

Another multisubunit RING E3 ubiquitin ligase is the APC/C. The APC/C regulation is dependent on 2 co-activators: Cdc20 during metaphase-anaphase transition and Cdh1 during mitotic exit and early G1 phase [136]. When the APC/C is activated by Cdc20, cell cycle proteins such as securin and cyclin B are targeted for degradation by the proteasome leading to onset of the anaphase [137]. We showed based on gene expression analysis that the co-activator Cdc20 is highly expressed in high-risk MM patients. Moreover, high Cdc20 expression correlated with poor prognosis in MM patients. The APC/C inhibitor proTAME induced an accumulation of the APC/C^cdc20^ substrate cyclin B1 in MM cells and caused MM cells to accumulate in metaphase. Moreover proTAME induced a significant dose-dependent decrease in viability and increase in apoptosis in MM cells. The induction of apoptosis was associated with caspase 3, 8, 9 and PARP cleavage. Moreover, we demonstrated that the observed cell death was due to accumulation of the pro-apoptotic molecule Bim. The combination of proTAME and another APC/C inhibitor, apcin, or melphalan resulted in an increased anti-MM activity. Our study suggests that the APC/C and its co-activator Cdc20 could be a novel and promising target especially in high-risk MM patients (unpublished results).

### Deubiquitinating enzymes (DUBs)

The ubiquitination process can be reversed by a group of proteases called DUBs, which recognize ubiquitinated proteins and remove their ubiqtuitin tags by cleavage of the isopeptide bond at the C-terminus of ubiquitin [138]. At the moment, 79 functional DUBs with 5 different subfamilies have been identified in the human genome. Most of the subfamilies are cysteine proteases and one of the families consists of zinc metalloproteases [139]. DUBs are considered to have three major functions: processing inactive ubiquitin precursors, removing ubiquitin chains from modified substrates to prevent proteasomal degradation and editing of ubiquitin chains [140]. DUBs are direct antagonists of E3 ubiquitin ligases and are increasingly suggested as potential targets in various malignancies [141]. In MM, it has been shown that high expression of the DUB Usp9x is correlated with poor prognosis and is proposed to be involved in the stabilization of the survival protein, Mcl-1 [142]. Moreover in another study they demonstrated that the partially selective Usp9x inhibitor WP1130 induced apoptosis and reduced Mcl-1 levels in human MM cells. When Uspx9 was depleted with shRNA, transient induction of apoptosis was achieved followed by a sustained cell growth inhibition. Remarkably the closely related Usp24 was upregulated when Usp9x was depleted. Depletion of Usp24 did result in significant induction of apoptosis and reduction in Mcl-1 levels in MM cells. Usp24 was able to sustain myeloma survival and regulate Mcl-1 in the absence of Usp9x. Interestingly both Usp9x and Usp24 were expressed and activated in primary MM cells. A more novel inhibitor EOAI3402143 inhibited Usp9x and Usp24 activity in a dose-dependent manner and resulted in the induction of apoptosis. Importantly, EOAI3402143 was able to suppress tumor growth *in vivo* [143]. Another DUB that has been shown to be highly expressed and correlated with poor overall survival in MM is Usp7. Inhibition of Usp7 with the selective inhibitor P5091 induces apoptosis in MM cells resistant to conventional therapy and acts synergistically with the HDAC inhibitor SAHA, lenalidomide and dexamethasone. The underlying mechanism of the induced cell death was attributed to the activation of the HDM2/p53/p21 signaling axis. Usp7 deubiquitinates and stabilizes the E3 ubiquitin ligase HDM2 leading to increased degradation of p53. P5091 was potent in inhibiting tumor growth and prolonged survival in a xenograft mouse model [144]. These studies suggest that Usp9x, Usp24 and Usp7 could be potential new therapeutic targets in MM. Moreover it gives the rationale for further clinical investigation of these compounds, alone or in combination, for the treatment of MM.

A summary of the compounds targeting the different components of the UPS and their current clinical status can be found in Table 1. Moreover Figure 2 illustrates the interference of each compound in the UPS pathway.

**Table 1:** pharmacological agents targeting the ubiquitin proteasome system

Drug	Molecular target	Status
**Proteasome/boronates**		
Bortezomib	20S proteasome (chymotrypsin activity)	FDA approved for MM
Ixazomib	20S proteasome (chymotrypsin activity)	Phase 3
Delanzomib	20S proteasome (chymotrypsin activity)	Phase 1/2
**Proteasome/epoxyketones**		
Carfilzomib	20S proteasome (chymotrypsin activity)	FDA approved for MM
Oprozomib	20S proteasome (chymotrypsin activity)	Phase 1/2
**Proteasome/salinosporamides**		
Marizomib	20S proteasome (chymotrypsin, trypsin, caspase activity)	Phase 1/2
**E1 ubiquitin activating enzyme**		
PYR-41	UBA1	Preclinical (not in MM)
NSC624206	UBA1	Preclinical (not in MM)
JS-K	UBA1	Preclinical
PYZD-4409	UBA1	Preclinical
**NEDD8 activating enzyme**		
MLN4924	NAE	Phase 1
**E2 conjugating enzyme**		
CC0651	CDC34	Preclinical (not in MM)
**E3 ubiquitin ligase/p53 potentiators**		
Nutlin-3	HDM2	Preclinical
RITA	HDM2	Preclinical
MI-63	HDM2	Preclinical
Serdemetan	HDM2	Phase 1 (not for MM)
**E3 ubiquitin ligase/smac mimetics**		
LBW242	IAPs	Preclinical
LCL161	IAPs	Phase 2
AT-406	IAPs	Phase 1 (not for MM)
**E3 ubiquitin ligase/others**		
Compound A	SCF^Skp2^	Preclinical
IMiDs	Cereblon	FDA approved for MM
proTAME	APC/C	Preclinical
**Deubiquitinating enzyme**		
WP1130	Usp9x	Preclinical
EOAI3402143	Usp9x and Usp24	Preclinical
P5091	Usp7	Preclinical

## CONCLUDING REMARKS AND FUTURE PERSPECTIVES

The UPS consists of ubiquitin, ubiquitin-activating enzymes, ubiquitin-conjugating enzymes, ubiquitin ligases, deubiquitinases and the proteasome. All of these components are involved in the pathogenesis of MM and are therefore considered as potential therapeutic targets in MM. The success of bortezomib in the treatment of MM sets the precedent for this direction. A second generation of proteasome inhibitors is currently under development and clinical investigation with the ambition to increase efficacy, enhance anti-MM activity, decrease toxicity, increase the flexibility of dosing and improve convenience for patients. Preclinical and clinical studies with second generation proteasome inhibitors are promising and are certainly warranted for further studies. Since the proteasome is also essential in normal cells, scientists are now investigating upstream enzymes of the UPS that are aberrantly expressed in MM. This will affect fewer proteins and could potentially lead to less toxicity. Most of the agents targeting components upstream of the proteasome are still under preclinical research, while a few are currently under clinical investigation for the treatment of MM or other malignancies. Advances in our knowledge of the role of the different components of the UPS in the pathogenesis of MM are necessary. Moreover great efforts should be put in the optimization and development of new molecules targeting UPS components. Persistent research in this area will certainly lead to an improvement in the treatment of MM patients.
